# Characterizing tau propagation using a chronic injection model in a C57BL/6 mouse hippocampus

**DOI:** 10.1002/alz70855_099171

**Published:** 2025-12-23

**Authors:** Daniel Lamontagne‐Kam, Arsalan Rahimabadi, Dainelys Guadarrama Bello, Marc Lavallée‐Beaulieu, Aseel El Fermawi, Laurie Bonenfant, Antonio Nanci, Habib Benali, Jonathan Brouillette

**Affiliations:** ^1^ Université de Montréal, Montréal, QC, Canada; ^2^ Concordia University, Montreal, QC, Canada; ^3^ PERFORM Centre, University of Concordia, Montreal, QC, Canada

## Abstract

**Background:**

Tau pathology is an important neuropathological marker of Alzheimer's Disease (AD) and correlates closely with neurodegeneration and cognitive decline. To date, much of the work examining tau propagation has been performed using mutated tau and/or transgenic animal models. Since tau is not mutated in AD, the main objective of this study is to characterize the propagation of non‐mutated tau in a mouse model.

**Methods:**

Tau preformed fibrils (2 µg) or a vehicle solution were injected in the hippocampus of 2‐month‐old C57BL/6J mice. Tau propagation was evaluated at different time points following single or chronic injections (24 h after one injection, or 24 h, 1, 5, 9 or 13 weeks after 5 consecutive days of injections).

**Results:**

Patterns of propagation and neurodegeneration were determined using a variety of fluorescent antibodies and confocal microscopy. Images of positive controls show tau hyperphosphorylation at the CA1 region of the hippocampus, as expected. Images from treatment groups will be compiled to form a 3D model of tau propagation over time.

**Conclusions:**

Examining the propagation of non‐mutated fibrils of human tau in a C57BL/6 wild‐type mouse model will provide novel information regarding how tau can influence neurodegeneration and cognitive decline in the early stages of AD.

